# Age, corticosteroid treatment and site of mutations affect motor functional changes in young boys with Duchenne Muscular Dystrophy

**DOI:** 10.1371/journal.pone.0271681

**Published:** 2022-07-29

**Authors:** Giorgia Coratti, Jacopo Lenkowicz, Giulia Norcia, Simona Lucibello, Elisabetta Ferraroli, Adele d’Amico, Luca Bello, Elena Pegoraro, Sonia Messina, Federica Ricci, Tiziana Mongini, Angela Berardinelli, Riccardo Masson, Stefano C. Previtali, Grazia D’angelo, Francesca Magri, Giacomo P. Comi, Luisa Politano, Luigia Passamano, Gianluca Vita, Valeria A. Sansone, Emilio Albamonte, Chiara Panicucci, Claudio Bruno, Antonella Pini, Enrico Bertini, Stefano Patarnello, Marika Pane, Eugenio Mercuri, for the italian DMD study group

**Affiliations:** 1 Pediatric Neurology, Department of Woman and Child Health and Public Health, Child Health Area, Università Cattolica del Sacro Cuore, Rome, Italy; 2 Centro Clinico Nemo, Fondazione Policlinico Universitario Agostino Gemelli IRCCS, Rome, Italy; 3 Fondazione Policlinico Universitario A.Gemelli IRCCS, Università Cattolica del Sacro Cuore, Rome, Italy; 4 Department of Neurosciences, Unit of Neuromuscular and Neurodegenerative Disorders, Bambino Gesù Children’s Hospital, Rome, Italy; 5 Department of Neurosciences, University of Padua, Padua, Italy; 6 Department of Clinical and Experimental Medicine, University of Messina, Messina, Italy; 7 Neuromuscular Center, AOU Città della Salute e della Scienza, University of Turin, Torino, Italy; 8 IRCCS Mondino Foundation, Pavia, Italy; 9 Fondazione IRCCS Istituto Neurologico Carlo Besta, Milan, Italy; 10 Division of Neuroscience, IRCCS San Raffaele Scientific Institute, Milan, Italy; 11 Scientific Institute IRCCS E. Medea, Bosisio Parini, Italy; 12 Dino Ferrari Centre, Department of Pathophysiology and Transplantation, Fondazione IRCCS Ca’ Granda Ospedale Maggiore Policlinico, University of Milan, Milan, Italy; 13 Cardiomyology and Medical Genetics, University of Campania Luigi Vanvitelli, Naples, Italy; 14 The NEMO Center in Milan, Neurorehabilitation Unit, ASST Niguarda Hospital, University of Milan, Milan, Italy; 15 Center of Translational and Experimental Myology, IRCCS Istituto Giannina Gaslini, and Department of Neuroscience, Rehabilitation, Ophtalmology, Genetics, Maternal and Child Health—DINOGMI, University of Genova, Genoa, Italy; 16 Neuromuscular Pediatric Unit, UOC di Neuropsichiatria dell’età pediatrica, IRCCS Istituto delle Scienze Neurologiche di Bologna, Bologna, Italy; Emory University, UNITED STATES

## Abstract

The aim of this study was to establish the possible effect of age, corticosteroid treatment and brain dystrophin involvement on motor function in young boys affected by Duchenne Muscular Dystrophy who were assessed using the North Star Ambulatory Assessment between the age of 4 and 7 years. The study includes 951 North Star assessments from 226 patients. Patients were subdivided according to age, to the site of mutation and therefore to the involvement of different brain dystrophin isoforms and to corticosteroids duration. There was a difference in the maximum North Star score achieved among patients with different brain dystrophin isoforms (p = 0.007). Patients with the involvement of Dp427, Dp140 and Dp71, had lower maximum NSAA scores when compared to those with involvement of Dp427 and Dp140 or of Dp427 only. The difference in the age when the maximum score was achieved in the different subgroups did not reach statistical significance. Using a linear regression model on all assessments we found that each of the three variables, age, site of mutation and corticosteroid treatment had an influence on the NSAA values and their progression over time. A second analysis, looking at 12-month changes showed that within this time interval the magnitude of changes was related to corticosteroid treatment but not to site of mutation. Our findings suggest that each of the considered variables appear to play a role in the progression of North Star scores in patients between the age of 4 and 7 years and that these should be carefully considered in the trial design of boys in this age range.

## Introduction

Over the last few years there has been increasing attention to the early patterns of changes observed in boys affected by Duchenne Muscular Dystrophy (DMD). A few studies have reported the use of the North Star Ambulatory Assessment (NSAA) in boys as young as three years, identifying the items that are more likely to be affected by development [1–4]. By the age of 4 years all the items in the NSAA are performed by typically developing children and the whole scale can therefore be reliably used to assess changes over time [2]. Between the age of 4 and 7 years there is a progressive increase in the mean NSAA score, but with some variability in the changes as not all the boys show an improvement, with some patients remaining more stable and others showing signs of decline [5–7].

The variability has been reported as possibly due to a number of variables [8]. Most DMD patients start corticosteroid treatment (CS) between the age of 4 and 6 years and this may boost the possibility of an improvement. There is also increasing evidence that the site of mutation and the involvement of different brain dystrophin isoforms may affect motor performance and its progression [9–14].

Participants with DMD mutations involving the region upstream of intron 44 have involvement of Dp427 only, those with mutations involving the region from exon 51 to exon 62 have involvement of both Dp427 and Dp140 but not of Dp71 while those with mutations involving exon 63 and/or the region downstream of exon 63 have involvement of all three (Dp427/Dp140/Dp71). Patients with involvement of all three isoforms are more likely to achieve lower performances on the NSAA(8) and to develop global developmental delay [15], acquiring motor skills at a later age than those with the involvement of Dp427 and Dp140 or of Dp427 only.

The increased number of clinical trials specifically targeting young DMD boys between 4 and 7 years of age has highlighted the need to better understand the variability of changes in order to set up the right expectations and facilitate trial design in this age group.

The aim of this study was to establish the possible effect of a number of variables, including age, CS treatment and brain dystrophin involvement on the patterns of changes observed in young DMD boys. More specifically, we wished to see whether these changes could be described by a multivariate model exploring the possible impact of different variables against the NSAA value. To do so, we also enabled interaction terms between the variables in the model and find out if one or more of them behaved differently when measured in different subgroups of patients.

## Material and methods

The study is a multicentric cohort study involving 13 tertiary neuromuscular centers in Italy. Patients were recruited between January 2004 and June 2021. The study was approved by the Ethical Committees of all the participating centers (Catholic University, Rome; Centro Clinico Nemo, University of Milano, Milan; IRCCS Eugenio Medea Bosisio-Parini, Bosisio-Parini; IRCCS Istituto Giannina Gaslini, Genoa; University of Messina, Messina; IRCCS Ospedale San Raffaele, Milan; Fondazione IRCCS Istituto Neurologico Besta, Milan; Fondazione IRCCS Ca’ Granda—Ospedale Maggiore Policlinico, Milan; University of Napoli, Naples; Ospedale Bambino Gesù, Rome; University of Padua, Padua; Istituto Mondino, Pavia; University of Turin, Turin; Neuromuscular Pediatric Unit, IRCCS Istituto delle Scienze Neurologiche di Bologna, Bologna). Written informed consent was obtained from all guardians of participants in the study.

Patient inclusion criteria at baseline were: clinical and genetically proven DMD diagnosis, no severe or moderate learning difficulties or behavioral problems, able to perform a valid NSAA [16]. All patients who had assessments between the age of 4 and 7 years who fulfilled the inclusion criteria were enrolled in the study.

### NSAA

The scale consists of 17 items, ranging from standing (item 1) to running (item 17) and includes several items assessing abilities that are necessary to remain functionally ambulant, items assessing abilities, such as head raise and standing on heels that can be partly present in the early stages of the disease and a number of activities such as hopping, or running [16].

Each item can be scored on a 3-point scale using simple criteria: 2 -Normal achieves goal without any assistance; 1 -Modified method but achieves goal independent of physical assistance from another person; 0—Unable to achieve independently.

A total score can be achieved by summing the scores for all the individual items. The score can range from 0, if all the activities are failed, to 34, if all the activities are achieved.

The NSAA was performed by a physical therapist at each center, details of the training for the physiotherapists involved in the study and of the interobserver reliability for NSAA among the centers have already been reported [16,17].

### Statistical analysis

The study consists in 2 parts: a cross-sectional study, to understand the variance between patients, and a longitudinal study, assessing changes at 12 months (±3 months).

For the first endpoint of the analysis, the data were organized as follows: all patients with complete data were selected from the cross-sectional database and patients with a single visit were discarded. Descriptive statistics was conducted subdividing the population in age classes (4, 4.5, 5, 5.5, 6, 6.5, 7 years), according to the brain dystrophin involvement (Dp427 = mutations in exons <44; equivocal = 44–51; Dp140 = 52–63; Dp71 = >63) and to CS duration (no CS: never been on CS; <6 months: use of CS for less than 6 months; >6 months: at least 6 months of CS). Corticosteroid dosage and prescription was made on the basis of the recent recommendations [18].

Mean value and 95% Confidence interval/Standard deviation were reported as descriptive values for the different segments of the population. Analysis of variance (ANOVA) or Kruskal-Wallis Test were conducted to examine the differences on maximum value of NSAA ever achieved and the age at maximum value according to the type of brain dystrophin involvement. If the highest NSAA score occurred in more than one visit, the earliest one was selected. Furthermore, the complete dataset with all the visits for the same cohort of patients was used as input to multivariate linear regression models to quantify the association of individual explicative variables, i.e. age at visit, brain dystrophin involvement and CS duration, on the current visit NSAA value. The statistical design of the analysis did not consider time correlation between multiple visits of the same patient, rather each visit was modeled independently from the others and the time dependence is included in the model through the variable “age at visit”.

Finally, for the second endpoint of the study, a longitudinal dataset with 12-months paired visits for the same cohort of patients was analyzed to quantify differences in 12-months NSAA changes among brain dystrophin involvement and age categories. Mean values and inter-quartile ranges were reported as descriptive statistics for the dataset. Summarized t-test were used to compare the 12-months NSAA changes distribution between the groups of patients divided by brain dystrophin involvement, age category and duration of CS treatment. Significance level for statistical tests was set at .05. All data processing steps and statistical analysis was performed in R version 4.0.2.

## Results

Applying the inclusion criteria, 951 assessments from 226 patients were included for the cross-sectional study. The NSAA total scores ranged between 18 and 28 (mean 22.4). Details on the population subdivided in brain dystrophin involvement and CS duration can be found in Table 1.

**Table 1:** Population characteristics subdivided by brain dystrophin involvement and corticosteroids.

		All	Naïve	CS <6 months	CS >6 months
All	N visits (N pats):	951 (226)	374 (156)	151 (129)	426 (182)
	Age mean (95% ci)	5.72 (1.88)	5.05 (1.72)	5.71 (1.44)	6.30 (1.31)
	Age min-max	3.76–7.25	3.76–7.16	4.11–7.25	3.79–7.25
	NSAA mean (95% ci)	22.37 (12.82)	20.15 (12.49)	21.64 (12.59)	24.58 (11.75)
	NSAA min-max	1–34	5–33	2–34	1–34
Dp427 (< 44)	N visits (N pats):	425 (100)	170 (66)	70 (59)	185 (83)
	Age mean (95% ci)	5.74 (1.89)	5.05 (1.74)	5.77 (1.42)	6.35 (1.24)
	Age min-max	3.76–7.25	3.76–7.16	4.16–7.25	4.58–7.25
	NSAA mean (95% ci)	23.77 (12.03)	21.98 (11.91)	23.16 (12.21)	25.64 (11.04)
	NSAA min-max	1–34	6–33	2–34	1–34
Equivocal (44–51)	N visits (N pats):	230 (55)	93 (40)	37 (31)	100 (42)
	Age mean (95% ci)	5.68 (1.87)	5.14 (1.77)	5.57 (1.78)	6.22 (1.35)
	Age min-max	3.76–7.25	3.76–7.02	4.11–7.19	4.48–7.25
	NSAA mean (95% ci)	21.31 (12.67)	18.57 (11.69)	20.00 (12.04)	24.34 (11.25)
	NSAA min-max	1–34	5–32	4–30	1–34
+Dp140 (52–63)	N visits (N pats):	253 (59)	94 (42)	38 (33)	121 (49)
	Age mean (95% ci)	5.72 (1.89)	4.96 (1.65)	5.75 (1.14)	6.30 (1.41)
	Age min-max	3.77–7.25	3.77–7.11	4.30–7.21	3.79–7.25
	NSAA mean (95% ci)	21.66 (13.07)	19.36 (12.69)	20.87 (12.93)	23.70 (12.23)
	NSAA min-max	5–34	6–33	5–31	5–34
+Dp71 (>63)	N visits (N pats):	43 (12)	17 (8)	6 (6)	20 (8)
	Age mean (95% ci)	5.68 (1.79)	5.03 (1.73)	5.67 (1.14)	6.24 (1.22)
	Age min-max	4.00–7.13	4.00–7.00	4.70–6.47	5.05–7.13
	NSAA mean (95% ci)	18.40 (13.44)	14.76 (9.62)	18.83 (12.85)	21.35 (12.04)
	NSAA min-max	8–33	8–25	10–26	9–33

### Maximum NSAA value and brain dystrophin involvement

**Fig 1:**
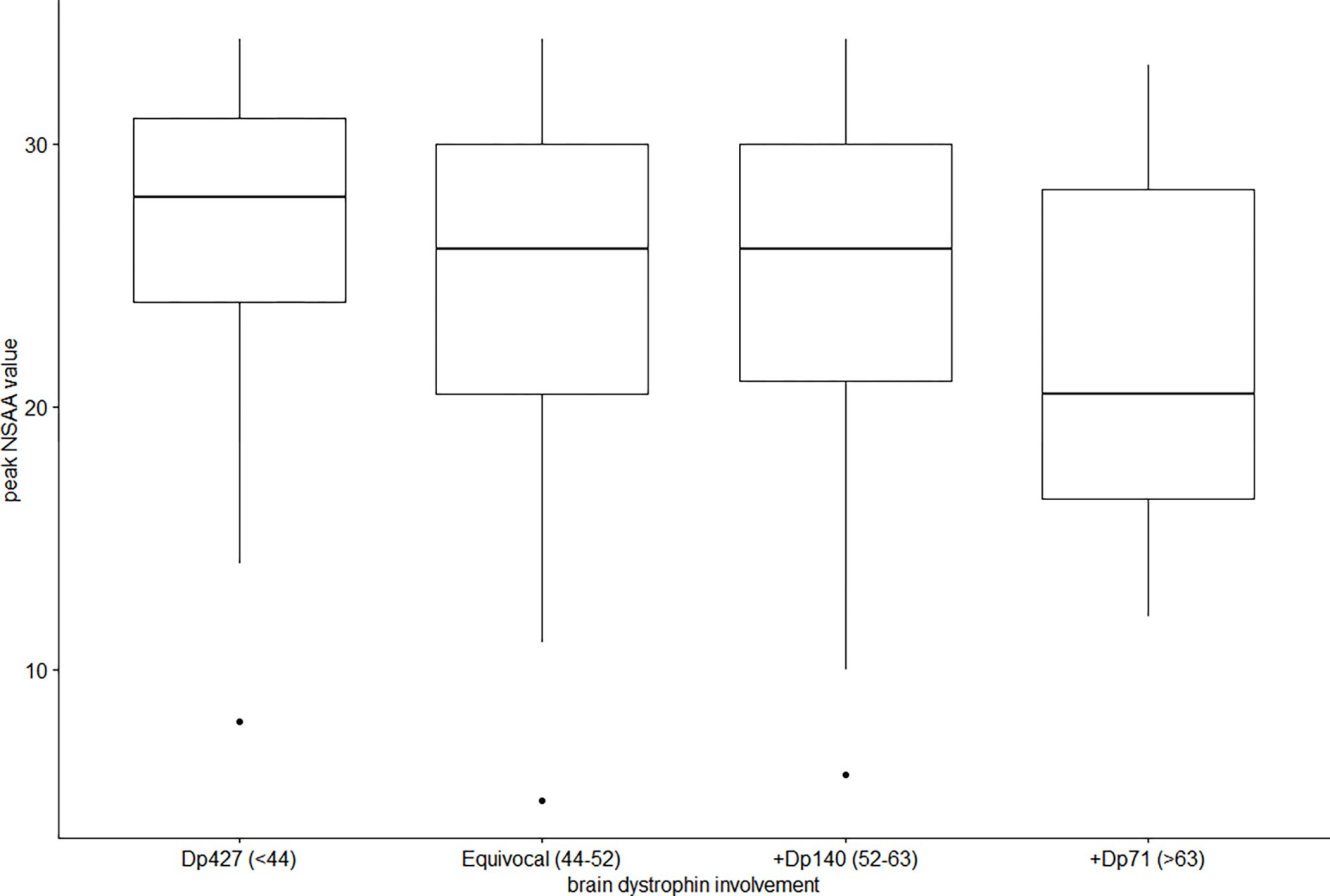
Maximum NSAA value and brain dystrophin involvement. Key to figure: Thick horizontal line = median value, box edges = 1^st^ and 3^rd^ quartile.

To determine the relationship between maximum NSAA value and site of mutation and therefore the involvement of different brain dystrophin isoforms, for each patient we retained the assessment corresponding to the first time when the maximum NSAA score was obtained, for a total of 226 assessments (Table 2). Kruskal-Wallis Test was conducted to examine the differences on maximum NSAA value according to the site mutation. Significant differences (Chi square = 12.0, P = .007, df = 3) were found among the four categories of participants (Fig 1, Table 2). A post hoc analysis was conducted via Dunn’s Kruskall Wallis test with Benjamini-Hochberg correction for multiple comparisons. The groups of brain dystrophin involvement which are significantly different in the post hoc analysis after p-value corrections are Dp427 vs +Dp71 (P = 0.039).

**Table 2:** Patient’s NSAA peak visit in the whole population and subgroups divided by brain dystrophin involvement and corticosteroids duration.

		All	Naïve	CS <6 months	CS >6 months
All	N:	226	56	33	137
	Age mean (95% ci)	6.05 (1.53)	5.42 (1.70)	6.12 (1.02)	6.30 (1.27)
	Age min—max	3.77–7.25	3.77–7.02	4.93–7.03	4.77–7.25
	NSAA mean (95% ci)	25.54 (11.70)	22.41 (14.68)	24.12 (10.99)	27.17 (9.12)
	NSAA min—max	5–34	5–33	12–34	16–34
Dp427 (< 44)	N:	100	22	17	61
	Age mean (95% ci)	6.18 (1.44)	5.56 (1.57)	6.17 (1.14)	6.41 (1.22)
	Age min—max	4.00–7.25	4.00–7.00	4.93–7.03	4.86–7.25
	NSAA mean (95% ci)	27.03 (9.87)	25.68 (11.51)	26.24 (9.98)	27.74 (9.09)
	NSAA min—max	8–34	8–33	14–34	17–34
Equivocal (44–51)	N:	55	15	6	34
	Age mean (95% ci)	6.01 (1.71)	5.45 (2.13)	6.10 (0.59)	6.24 (1.35)
	Age min—max	4.21–7.25	4.21–7.02	5.47–6–97	4.90–7.25
	NSAA mean (95% ci)	24.58 (12.12)	19.93 (14.54)	24.00 (7.54)	26.74 (9.25)
	NSAA min—max	5–34	5–32	20–30	16–34
+Dp140 (52–63)	N:	59	15	8	36
	Age mean (95% ci)	5.94 (1.47)	5.26 (1.48)	6.04 (0.59)	6.20 (1.25)
	Age min—max	3.77–7.14	3.77–6.51	5.78–6.65	4.77–7.14
	NSAA mean (95% ci)	24.73 (12.64)	22.00 (16.81)	20.88 (12.85)	26.72 (8.82)
	NSAA min—max	6–34	6–33	12–31	18–34
+Dp71 (>63)	N:	12	4	2	6
	Age mean (95% ci)	5.79 (1.59)	5.21 (1.73)	6.01 (-)	6.09 (1.39)
	Age min—max	4.51–7.13	4.51–6.50	5.56–6.47	5.16–7.13
	NSAA mean (95% ci)	21.58 (13.54)	15.25 (5.63)	19.50 (-)	26.50 (11.55)
	NSAA min—max	12–33	12–19	17–22	17–33

### Age at maximum NSAA value and brain dystrophin involvement

**Fig 2:**
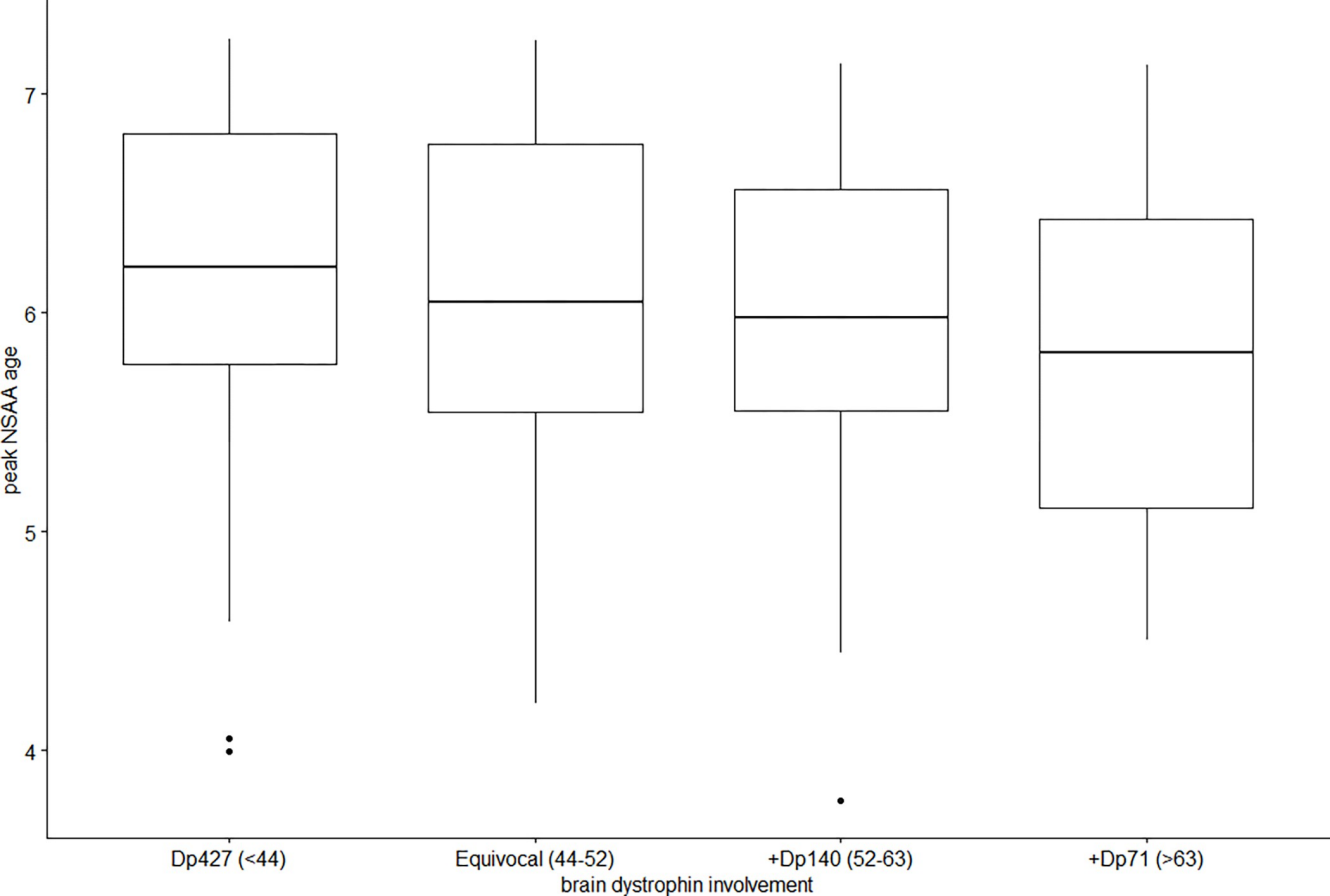
Age at maximum NSAA value and brain dystrophin involvement. Key to figure: Thick horizontal line = median value, box edges = 1^st^ and 3^rd^ quartile.

Kruskal-Wallis Test was conducted to analyze the differences on age at maximum value of NSAA achieved according to the brain dystrophin involvement. No significant differences (Chi square = 5.4, P = .14, df = 3) were found among the four categories of participants (Fig 2, Table 2).

### Influence of corticosteroid treatment, age and brain dystrophin involvement on NSAA value

Multivariate linear regression models were employed to quantify the association of individual variables, i.e. age at visit, brain dystrophin involvement and CS duration, on the current visit NSAA value. In a first model the CS duration variable was considered as categorical with three categories (no CS, CS from less than 6 months, CS from more than 6 months). The 6 month period was chose in agreement with the inclusion criteria of many clinical trials that request at least 6 month of CS as a more marked functional improvement may be observed in the first months after the therapy is started. The contribution of CS category less than 6 months was not statistically significant (P = 0.3). As the CS category less than 6 months was not statistically different from that of no CS, and both were not contributing to NSAA values, a second linear regression model was estimated using a binary variable for the CS duration: more than 6 months compared to other.

Finally, two other liner regression models were used to estimate the impact of the interaction terms between age and brain dystrophin involvement and the impact of the interaction between both age and brain dystrophin involvement with age and CS duration. All models were selected through an AIC based stepwise selection procedure. The most complete model included two interaction terms: one between age and CS duration, and the other between age and brain dystrophin involvement. The most complete model’s coefficients and p-values are reported in Table 3. According to the model coefficients and all else being equal, the NSAA increases on average 1.86 points each year for all the brain dystrophin involvement subgroups except the Dp71, which starts lower than the other subgroups, but has higher increase rate per unit age due to the positive interaction term between the variables. With regard to the CS status, being in treatment since more than 6 months has a positive impact on the predicted NSAA value, but this effect decreases with age due to the negative coefficient of the interaction term between the variables.

**Table 3:** Coefficients, standard errors, and p-values for the explanatory variables in the complete model.

Variable	Coefficient	Standard error	P-value
Intercept	12.1	1.5	p < 0.0001
Age (years)	1.86	0.28	p < 0.0001
Equivocal	-2.20	0.48	p < 0.0001
+Dp140	-2.05	0.46	p < 0.0001
+Dp71	-18.6	5.80	P = 0.001
Duration of CS treatment more than 6 months	10.7	3.09	P = 0.0005
Interaction term: Age—Duration of CS treatment more than 6 months	-1.39	0.51	P = 0.006
Interaction term: Age—+Dp71	2.39	1.00	P = 0.02

**Fig 3:**
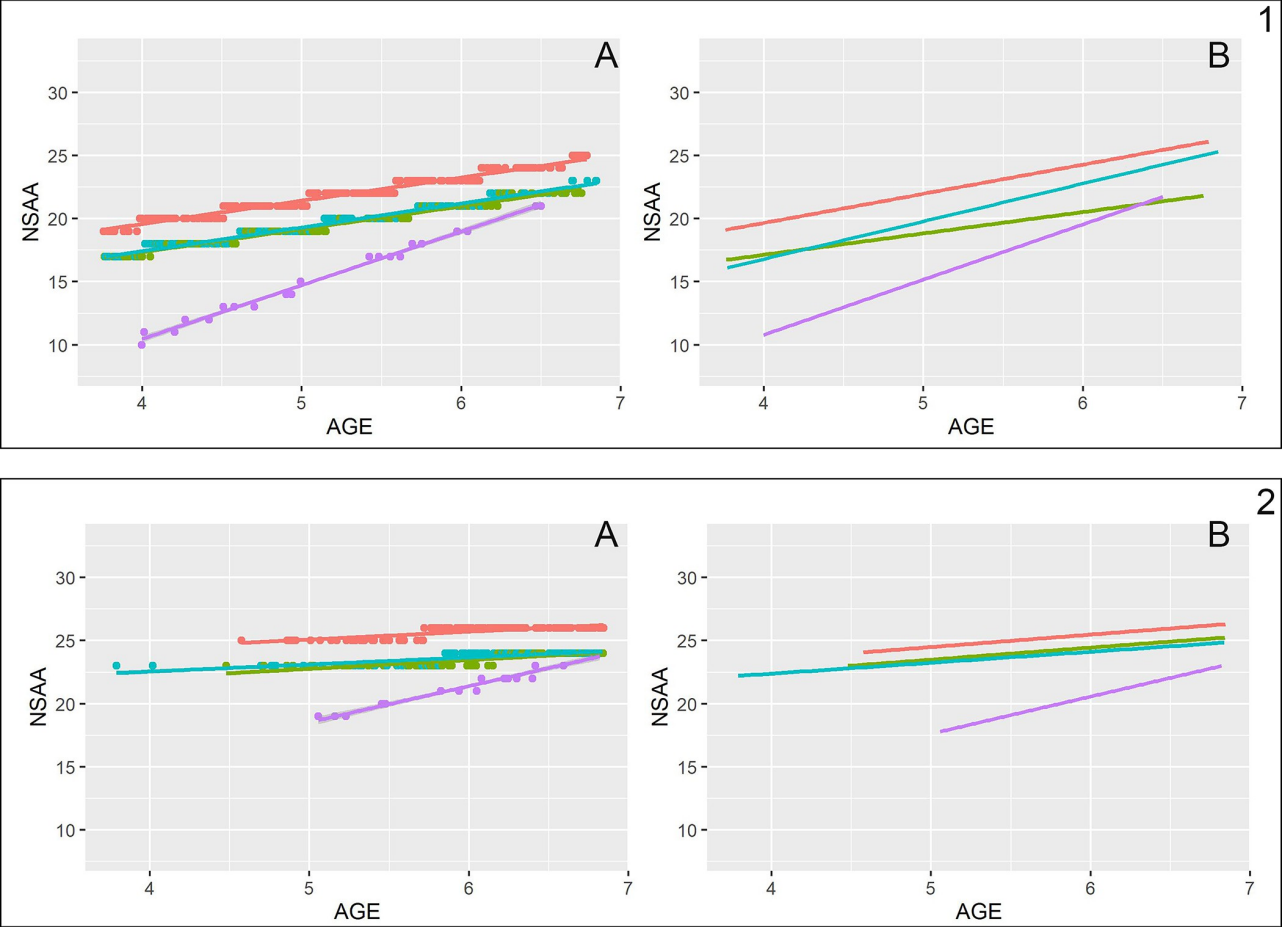
Linear fit on modeled vs real data by brain dystrophin involvement and corticosteroid status. Key to figure: Panel 1 = Patients on CS treatment on less than 6 months/naive, (A) modeled (B) real; Panel 2 = Patients on CS treatment on less from more than 6 months, (A) modeled (B) real. Color coding: Red = Dp427, green = Equivocal, blue = Dp140, purple = Dp71.

Fig 3 shows a comparison between the data generated with this last model and the corresponding mean trajectories from real data.

#### 12-month changes

Of the 226 patients included in the cross-sectional analysis, 196 also had at least one pair of assessments at 12 months. Seventy-three of the 196 patients had only one paired assessment, 49 had 2, 74 had more than 2, for a total of 468 paired assessments at 12 months. At 12 months, the mean changes on all the paired assessments was +1.67 (CI = 1.38).

When analyzing the paired assessments according to CS treatment, the mean 12 month change in patients who had no treatment was 0.98 (CI = 7.55) and was significantly different from the mean change in the assessments from boys on CS treatment (1.87 (CI = 7.95)) (p = 0.037). In particular, it was significant against the group who had CS for less than 6 months (2.07 (CI = 8.13)) (p = 0.04) but not from the mean change (1.78 (CI = 7.85)) who followed the treatment from more than 6 months (p = 0.08).

When analyzing the paired assessments according to type of mutation, there was no difference among subgroups.

Table 4 shows changes subdivided in classes according to brain dystrophin involvement. More details can be found in S1 Table.

**Table 4:** Summary of baseline characteristics at baseline, 12-month, and changes over 12 months, subdivided by brain dystrophin involvement.

			NSAA		
Whole cohort		AGE AT BASELINE	BASELINE	12 MONTHS	12-MONTH CHANGE
**All (n:468)**	**mean (95%CI)**	5.19(1.38)	21.96(11.71)	23.63(12.18)	1.67(7.89)
	**min-max**	3.76;6.25	4;34	1;34	-10;15
**Dp427 (n:209)**	**mean (95%CI)**	5.19(1.41)	23.14(11.24)	24.83(11.89)	1.69(7.92)
	**min-max**	3.76;6.22	6;34	2;34	-10;12
**Equivocal (n:100)**	**mean (95%CI)**	5.19(1.31)	21.16(11.34)	23.03(11.39)	1.87(7.81)
	**min-max**	3.89;6.24	4;34	1;34	-9;11
**+Dp140 (n:128)**	**mean (95%CI)**	5.16(1.42)	21.42(11.88)	22.89(12.38)	1.47(8.18)
	**min-max**	3.77;6.25	6;33	5;34	-8;15
**+Dp71 (n:24)**	**mean (95%CI)**	5.31(1.33)	18.67(12.75)	20.29(12.68)	1.62(7.07)
	**min-max**	4;6.25	8;30	11;33	-3;9

## Discussion

Until recently many clinical trials in DMD have mainly included patients above the age of 7 years [19,20], due to evidence from natural history studies of a higher risk of decline in untreated patients above the age of 7 years when compared to younger patients who, in contrast, are more stable or even show some improvement. The possibility of an improvement in younger children has also been confirmed by using more advanced statistical approaches identifying trajectories of progression [21,22]. Despite the overall increase in mean scores individual boys may present with different trends of progression, from small improvements to stability and, in some cases, even with some decline in scores [23]. Recent clinical trials focusing on young DMD boys have highlighted the need for a better understanding of the variability of changes before the age of 7 years as, especially when dealing with relatively small numbers, as the results may be affected by inclusion criteria and randomization.

A number of variables have been considered to potentially influence the patterns of changes in this age group. CS are not only known to delay the overall progression of the disease [24,25] but in the first 6 to 12 months after they are started, they are frequently associated with some functional improvement. Recent studies highlighted how the site of mutation and the involvement of different dystrophin isoforms may also affect the scores on developmental scales [15,26], early milestones [27] and NSAA scores [3].

The aim of this study was to use a systematic approach to establish the possible effect of these variables on the NSAA scores by subdividing the cohort according to age, site of mutation and CS treatment. In agreement with recent findings [8], we also found that patients with mutations affecting more dystrophin isoforms (Dp427, Dp140 and Dp71) achieved lower maximum scores compared to those with mutations in the first part of the gene in whom only Dp427 was involved (21.6 vs 27.0). Patients with mutations affecting Dp427 and Dp140 but not Dp71 had intermediate scores. Intermediate scores were also found in patients with mutations between exon 44 and 51 in whom the definition of brain dystrophin involvement is ambiguous and were excluded from the previous recent study.

We also confirmed that while there was a difference in the age when the maximum score in each category was achieved, this did not reach statistical significance. These findings confirm the hypothesis that DMD boys with involvement of Dp427, Dp140 and Dp71, who are known to be at higher risk of cognitive impairment, have a general neurodevelopmental delay that leads to a delay in achieving some milestones or activities, with a progressive increase in NSAA scores over time.

The use of a linear regression model allowed to provide additional information, confirming the hypothesis that each of the three variables considered in the analysis, i.e. age, site of mutation and CS treatment had an influence on the NSAA values and their progression over time. In this age group the older patients, not being on CS and having mutations affecting Dp 71 had a higher risk of lower scores and more rapid changes. When we assessed the interaction between individual factors we found that the NSAA progression was strongly influenced by age and CS treatment, with a negative effect of each year of age to the positive effect of CS treatment (-1.39/year, p = 0.006). The interaction between age and site of mutation confirmed that patients with mutations affecting Dp71 had a different trend of progression compared to the other subgroups, as they had lower NSAA scores at a younger age, and gained more points /year in comparison to the whole cohort before reaching their maximum score.

The analysis of 12 month changes confirmed that, as previously reported in other natural history studies, before the age of 7 years there is an overall mean improvement in the NSAA scores. In the present paper we also observed that the magnitude of changes was partly related to CS treatment but not to site of mutation. The mean positive changes in the subgroups with different dystrophin isoforms were all in a narrow range, even if their baseline NSAA scores were different. Corticosteroid treatment, in contrast, was more relevant as patients with no CS treatment had lower changes compared to both those on treatment over 6 months and to those who had started the treatment within 6 months who showed the highest changes.

These results suggest that each of the considered variables appear to play a role in the progression of NSAA scores in patients between the age of 4 and 7 years: patients with mutations also affecting Dp71 in particular, appear to have a distinct pattern of NSAA scores over time. These findings suggest that attention should be paid at the time of recruiting boys in clinical trials and that the possible effect of the variables assessed in the present study should be carefully considered at the time of selecting inclusion and randomization criteria.

## Supporting information

S1 Table Age and NSAA changes for the whole population and by brain dystrophin involvement and age subgroups. (DOCX)
