# Supplementary material for: Age, corticosteroid treatment and site of mutations affect motor functional changes in young boys with Duchenne Muscular Dystrophy
Source: PLoS One. 2022 Jul 29;17(7):e0271681. doi: 10.1371/journal.pone.0271681 (PMC9337636; doi:10.1371/journal.pone.0271681)
Supplement: S1 Table — (DOCX) [file pone.0271681.s001.docx]

| **All** | | | | **AGE** | **NSAA T0** | **NSAA T12** | **NSAA CHANGE 12M** |
| --- | --- | --- | --- | --- | --- | --- | --- |
| All (n:468) | mean (95% CI) | | | 5.19(1.38) | 21.96(11.71) | 23.63(12.18) | 1.67(7.89) |
|  | min-max | | | 3.76;6.25 | 4;34 | 1;34 | -10;15 |
| 4-5years (n:63) | mean (95% CI) | | | 4.01(0.31) | 17.48(11.29) | 20.51(11.49) | 3.03(7.87) |
|  | min-max | | | 3.76;4.25 | 6;33 | 7;34 | -10;12 |
| 4,5-5,5years (n:75) | mean (95% CI) | | | 4.51(0.31) | 19.41(10.34) | 23(10.72) | 3.59(7.49) |
|  | min-max | | | 4.26;4.76 | 4;30 | 1;33 | -3;15 |
| 5-6years (n:89) | mean (95% CI) | | | 5.02(0.27) | 22.02(9.62) | 24.31(10) | 2.29(7.31) |
|  | min-max | | | 4.77;5.25 | 11;34 | 8;33 | -8;12 |
| 5,5-6,5 years (n:105) | mean (95% CI) | | | 5.49(0.29) | 23.4(11.28) | 24.48(12.79) | 1.08(7.94) |
|  | min-max | | | 5.26;5.75 | 9;34 | 7;34 | -9;12 |
| 6-7 years (n:136) | mean (95% CI) | | | 5.99(0.29) | 24.29(11.06) | 24.32(13.22) | 0.03(7) |
|  | min-max | | | 5.76;6.25 | 6;33 | 2;34 | -9;7 |
| **All w/o steroids** | | | | **AGE** | **NSAA T0** | **NSAA T12** | **NSAA CHANGE 12M** |
| All (n:107) | mean (95% CI) | | | 4.72(1.38) | 20.91(12.07) | 21.89(12.04) | 0.98(7.55) |
|  | min-max | | | 3.76;6.12 | 8;33 | 7;33 | -10;10 |
| 4-5years (n:34) | mean (95% CI) | | | 3.98(0.29) | 17.88(10.74) | 19.76(12.06) | 1.88(8.01) |
|  | min-max | | | 3.76;4.25 | 8;29 | 7;30 | -10;9 |
| 4,5-5,5years (n:30) | mean (95% CI) | | | 4.49(0.31) | 20.23(9.85) | 22.68(10.25) | 2.45(6.75) |
|  | min-max | | | 4.26;4.75 | 8;28 | 9;29 | -3;10 |
| 5-6years (n:18) | mean (95% CI) | | | 5.01(0.27) | 22.05(12.02) | 22.32(11.96) | 0.26(5.05) |
|  | min-max | | | 4.78;5.24 | 11;33 | 8;33 | -5;5 |
| 5,5-6,5 years (n:9) | mean (95% CI) | | | 5.43(0.31) | 21.89(12.57) | 20.22(15.21) | -1.67(7.27) |
|  | min-max | | | 5.26;5.75 | 12;30 | 8;29 | -9;3 |
| 6-7 years (n:16) | mean (95% CI) | | | 5.97(0.2) | 26.75(10.58) | 25.31(10.94) | -1.44(7.26) |
|  | min-max | | | 5.77;6.12 | 14;33 | 13;32 | -9;6 |
| **All with steroids <6m** | | | | **AGE** | **NSAA T0** | **NSAA T12** | **NSAA CHANGE 12M** |
| All (n:136) | mean (95% CI) | | | 5.16(1.22) | 21.3(12.46) | 23.38(13.34) | 2.07(8.13) |
|  | min-max | | | 3.76;6.19 | 4;34 | 1;34 | -8;15 |
| 4-5years (n:14) | mean (95% CI) | | | 4.08(0.32) | 17(14.81) | 21.71(13.44) | 4.71(7.11) |
|  | min-max | | | 3.76;4.25 | 6;33 | 12;34 | 0;12 |
| 4,5-5,5years (n:24) | mean (95% CI) | | | 4.49(0.29) | 18.33(11.39) | 22.04(11.81) | 3.71(8.1) |
|  | min-max | | | 4.26;4.75 | 4;30 | 1;31 | -3;15 |
| 5-6years (n:30) | mean (95% CI) | | | 5(0.29) | 22.17(10.96) | 25.07(9.94) | 2.9(7.57) |
|  | min-max | | | 4.77;5.25 | 13;34 | 13;33 | -3;12 |
| 5,5-6,5 years (n:37) | mean (95% CI) | | | 5.51(0.29) | 23.11(10.88) | 24.27(13.76) | 1.16(7.07) |
|  | min-max | | | 5.26;5.74 | 12;31 | 7;33 | -7;9 |
| 6-7 years (n:31) | mean (95% CI) | | | 5.91(0.24) | 22.55(12.69) | 22.45(16.26) | -0.1(7.98) |
|  | min-max | | | 5.76;6.19 | 6;31 | 2;34 | -8;6 |
| **All with steroids >6m** | | | | **AGE** | **NSAA T0** | **NSAA T12** | **NSAA CHANGE 12M** |
| All (n:221) | mean (95% CI) | | | 5.43(1.23) | 22.86(10.86) | 24.64(11.15) | 1.78(7.85) |
|  | min-max | | | 3.77;6.25 | 8;34 | 7;34 | -9;13 |
| 4-5years (n:15) | mean (95% CI) | | | 4.01(0.31) | 17(9.25) | 21.07(7.89) | 4.07(6.97) |
|  | min-max | | | 3.77;4.25 | 8;24 | 11;26 | -3;10 |
| 4,5-5,5years (n:20) | mean (95% CI) | | | 4.57(0.32) | 19.45(9.82) | 24.65(9.82) | 5.2(6.95) |
|  | min-max | | | 4.26;4.76 | 9;28 | 15;33 | -2;13 |
| 5-6years (n:40) | mean (95% CI) | | | 5.04(0.27) | 21.9(7.25) | 24.7(8.75) | 2.8(7.5) |
|  | min-max | | | 4.79;5.25 | 12;28 | 14;33 | -8;10 |
| 5,5-6,5 years (n:59) | mean (95% CI) | | | 5.48(0.28) | 23.81(11.43) | 25.25(11.43) | 1.44(8.34) |
|  | min-max | | | 5.27;5.75 | 9;34 | 7;34 | -9;12 |
| 6-7 years (n:87) | mean (95% CI) | | | 6.03(0.29) | 24.45(10.37) | 24.8(12.41) | 0.36(6.52) |
|  | min-max | | | 5.76;6.25 | 11;33 | 7;33 | -9;7 |
|  | | | | | | | |
| **Dp427** | | | | **AGE** | **NSAA T0** | **NSAA T12** | **NSAA CHANGE 12M** |
| All (n:209) | mean (95% CI) | | | 5.19(1.41) | 23.14(11.24) | 24.83(11.89) | 1.69(7.92) |
|  | min-max | | | 3.76;6.22 | 6;34 | 2;34 | -10;12 |
| 4-5years (n:29) | mean (95% CI) | | | 4(0.29) | 19.1(12.09) | 21.93(12.63) | 2.83(8.5) |
|  | min-max | | | 3.76;4.21 | 6;33 | 7;34 | -10;9 |
| 4,5-5,5years (n:33) | mean (95% CI) | | | 4.5(0.29) | 20.42(8.97) | 24.15(9.9) | 3.73(6.41) |
|  | min-max | | | 4.26;4.74 | 11;30 | 12;33 | -3;9 |
| 5-6years (n:40) | mean (95% CI) | | | 5.01(0.28) | 23.65(9.34) | 25.88(9.94) | 2.23(7.27) |
|  | min-max | | | 4.77;5.25 | 11;34 | 8;33 | -5;12 |
| 5,5-6,5 years (n:44) | mean (95% CI) | | | 5.49(0.3) | 24.61(10.84) | 25.75(11.92) | 1.14(7.76) |
|  | min-max | | | 5.26;5.75 | 12;33 | 7;34 | -9;12 |
| 6-7 years (n:63) | mean (95% CI) | | | 6(0.27) | 25.06(10.71) | 25.21(13.01) | 0.14(7.73) |
|  | min-max | | | 5.76;6.22 | 8;33 | 2;33 | -9;7 |
| **Dp427 w/o steroids** | | | | **AGE** | **NSAA T0** | **NSAA T12** | **NSAA CHANGE 12M** |
| All (n:53) | mean (95% CI) | | | 4.7(1.41) | 22.38(11.4) | 23.38(12.28) | 1(8.7) |
|  | min-max | | | 3.76;6.08 | 8;33 | 7;33 | -10;9 |
| 4-5years (n:17) | mean (95% CI) | | | 3.96(0.26) | 19.82(9.83) | 22.12(12.1) | 2.29(9.96) |
|  | min-max | | | 3.76;4.15 | 8;29 | 7;30 | -10;9 |
| 4,5-5,5years (n:15) | mean (95% CI) | | | 4.47(0.29) | 21(8.48) | 23.93(10.41) | 2.93(7.08) |
|  | min-max | | | 4.26;4.73 | 13;28 | 12;29 | -3;9 |
| 5-6years (n:9) | mean (95% CI) | | | 5(0.3) | 24.33(12.04) | 24.11(13.7) | -0.22(4.88) |
|  | min-max | | | 4.78;5.23 | 11;33 | 8;33 | -3;3 |
| 5,5-6,5 years (n:3) | mean (95% CI) | | | 5.38(0.26) | 23(18.7) | 21.33(22.72) | -1.67(4.93) |
|  | min-max | | | 5.26;5.52 | 12;29 | 8;29 | -4;1 |
| 6-7 years (n:9) | mean (95% CI) | | | 5.94(0.19) | 27.33(10.14) | 24.78(12.19) | -2.56(7.97) |
|  | min-max | | | 5.77;6.08 | 15;33 | 13;32 | -9;6 |
| **Dp427 with steroids <6m** | | | | **AGE** | **NSAA T0** | **NSAA T12** | **NSAA CHANGE 12M** |
| All (n:63) | mean (95% CI) | | | 5.16(1.25) | 22.46(12.71) | 24.65(13.47) | 2.19(7.95) |
|  | min-max | | | 3.76;6.19 | 6;34 | 2;34 | -8;12 |
| 4-5years (n:6) | mean (95% CI) | | | 4.01(0.39) | 17.83(21.09) | 22.17(17.46) | 4.33(5.5) |
|  | min-max | | | 3.76;4.21 | 6;33 | 12;34 | 1;8 |
| 4,5-5,5years (n:11) | mean (95% CI) | | | 4.48(0.27) | 19.36(11.13) | 22.64(10.49) | 3.27(5.69) |
|  | min-max | | | 4.27;4.69 | 11;30 | 15;31 | -1;8 |
| 5-6years (n:16) | mean (95% CI) | | | 5.01(0.3) | 23.69(10.34) | 27.19(8.97) | 3.5(7.33) |
|  | min-max | | | 4.77;5.25 | 16;34 | 18;33 | -1;12 |
| 5,5-6,5 years (n:16) | mean (95% CI) | | | 5.53(0.29) | 24.38(10.85) | 25.25(14) | 0.88(8.03) |
|  | min-max | | | 5.26;5.72 | 12;31 | 7;33 | -7;9 |
| 6-7 years (n:14) | mean (95% CI) | | | 5.94(0.25) | 23.29(12.17) | 23.71(16.85) | 0.43(9.34) |
|  | min-max | | | 5.78;6.19 | 8;29 | 2;33 | -8;6 |
| **Dp427 with steroids >6m** | | | | **AGE** | **NSAA T0** | **NSAA T12** | **NSAA CHANGE 12M** |
| All (n:93) | mean (95% CI) | | | 5.48(1.21) | 24.03(9.89) | 25.77(10.19) | 1.74(7.39) |
|  | min-max | | | 3.88;6.22 | 12;33 | 7;34 | -9;12 |
| 4-5years (n:6) | mean (95% CI) | | | 4.07(0.23) | 18.33(6.86) | 21.17(10.77) | 2.83(6.49) |
|  | min-max | | | 3.88;4.21 | 14;22 | 11;26 | -3;6 |
| 4,5-5,5years (n:7) | mean (95% CI) | | | 4.57(0.32) | 20.86(6.54) | 27(5.77) | 6.14(3.83) |
|  | min-max | | | 4.26;4.74 | 18;27 | 24;33 | 4;9 |
| 5-6years (n:15) | mean (95% CI) | | | 5.02(0.26) | 23.2(6.59) | 25.53(8.11) | 2.33(7.36) |
|  | min-max | | | 4.86;5.25 | 18;28 | 16;33 | -5;8 |
| 5,5-6,5 years (n:25) | mean (95% CI) | | | 5.47(0.3) | 24.96(10.29) | 26.6(8.73) | 1.64(7.78) |
|  | min-max | | | 5.27;5.75 | 12;33 | 16;34 | -9;12 |
| 6-7 years (n:40) | mean (95% CI) | | | 6.04(0.27) | 25.18(10.14) | 25.82(11.79) | 0.65(6.72) |
|  | min-max | | | 5.76;6.22 | 15;33 | 7;33 | -9;7 |
|  | | | | | | | |
| **Equivocal** | | | | **AGE** | **NSAA T0** | **NSAA T12** | **NSAA CHANGE 12M** |
| All (n:100) | mean (95% CI) | | | 5.19(1.31) | 21.16(11.34) | 23.03(11.39) | 1.87(7.81) |
|  | min-max | | | 3.89;6.24 | 4;34 | 1;34 | -9;11 |
| 4-5years (n:11) | mean (95% CI) | | | 4.1(0.28) | 16.18(8.49) | 20.18(9.1) | 4(7.69) |
|  | min-max | | | 3.89;4.25 | 9;24 | 12;27 | -2;10 |
| 4,5-5,5years (n:19) | mean (95% CI) | | | 4.51(0.34) | 17.89(11.92) | 21.32(12.7) | 3.42(7.28) |
|  | min-max | | | 4.26;4.75 | 4;28 | 1;32 | -3;11 |
| 5-6years (n:23) | mean (95% CI) | | | 5.06(0.29) | 20.91(9.55) | 23.7(8.51) | 2.78(7.52) |
|  | min-max | | | 4.82;5.25 | 12;30 | 16;32 | -5;10 |
| 5,5-6,5 years (n:19) | mean (95% CI) | | | 5.49(0.27) | 23.58(11.15) | 23.89(12.8) | 0.32(8.27) |
|  | min-max | | | 5.27;5.73 | 12;34 | 8;34 | -9;6 |
| 6-7 years (n:28) | mean (95% CI) | | | 5.98(0.32) | 23.89(8.92) | 24.18(11.83) | 0.29(6.46) |
|  | min-max | | | 5.76;6.24 | 15;33 | 13;33 | -7;7 |
| **Equivocal w/o steroids** | | | | **AGE** | **NSAA T0** | **NSAA T12** | **NSAA CHANGE 12M** |
| All (n:18) | mean (95% CI) | | | 4.81(1.05) | 20.44(9.49) | 20.72(11.95) | 0.28(6.98) |
|  | min-max | | | 4.05;6 | 13;30 | 8;32 | -9;8 |
| 4-5years (n:3) | mean (95% CI) | | | 4.13(0.21) | 15.67(2.99) | 15.67(6.3) | 0(3.92) |
|  | min-max | | | 4.05;4.25 | 14;17 | 12;18 | -2;2 |
| 4,5-5,5years (n:6) | mean (95% CI) | | | 4.5(0.44) | 19.67(8.38) | 22.17(6.95) | 2.5(6.04) |
|  | min-max | | | 4.26;4.75 | 13;25 | 16;26 | -1;8 |
| 5-6years (n:6) | mean (95% CI) | | | 5.05(0.31) | 22.5(8.83) | 22.33(10.78) | -0.17(5.17) |
|  | min-max | | | 4.86;5.24 | 17;30 | 17;30 | -5;3 |
| 5,5-6,5 years (n:2) | mean (95% CI) | | | 5.43(0.37) | 19(5.54) | 13.5(15.25) | -5.5(9.7) |
|  | min-max | | | 5.29;5.56 | 17;21 | 8;19 | -9;-2 |
| 6-7 years (n:1) | mean (95% CI) | | | 6(NA) | 30(NA) | 32(NA) | 2(NA) |
|  | min-max | | | 6;6 | 30;30 | 32;32 | 2;2 |
| **Equivocal with steroids <6m** | | | | **AGE** | **NSAA T0** | **NSAA T12** | **NSAA CHANGE 12M** |
| All (n:26) | mean (95% CI) | | | 5.03(1.3) | 19.88(12.96) | 22.35(12.54) | 2.46(8.39) |
|  | min-max | | | 3.9;6.03 | 4;30 | 1;32 | -5;11 |
| 4-5years (n:4) | mean (95% CI) | | | 4.12(0.32) | 14.75(8.52) | 21(9.73) | 6.25(5.63) |
|  | min-max | | | 3.9;4.25 | 9;19 | 16;27 | 4;10 |
| 4,5-5,5years (n:7) | mean (95% CI) | | | 4.49(0.28) | 16.14(13.65) | 19.71(17) | 3.57(9.32) |
|  | min-max | | | 4.26;4.67 | 4;24 | 1;27 | -3;11 |
| 5-6years (n:4) | mean (95% CI) | | | 5.02(0.44) | 19.25(13.61) | 22.75(5.85) | 3.5(9.8) |
|  | min-max | | | 4.82;5.25 | 13;29 | 19;26 | -3;9 |
| 5,5-6,5 years (n:5) | mean (95% CI) | | | 5.53(0.18) | 25.8(7.39) | 26(12.32) | 0.2(5.07) |
|  | min-max | | | 5.44;5.67 | 20;29 | 17;32 | -3;3 |
| 6-7 years (n:6) | mean (95% CI) | | | 5.85(0.2) | 23.17(8.71) | 23(12.02) | -0.17(6.72) |
|  | min-max | | | 5.76;6.03 | 17;30 | 14;30 | -5;4 |
| **Equivocal with steroids >6m** | | | | **AGE** | **NSAA T0** | **NSAA T12** | **NSAA CHANGE 12M** |
| All (n:55) | mean (95% CI) | | | 5.38(1.25) | 21.95(11.09) | 24.04(10.36) | 2.09(7.72) |
|  | min-max | | | 3.89;6.24 | 9;34 | 13;34 | -7;10 |
| 4-5years (n:4) | mean (95% CI) | | | 4.04(0.33) | 18(11.54) | 22.75(6.28) | 4.75(8.06) |
|  | min-max | | | 3.89;4.25 | 12;24 | 18;25 | 1;10 |
| 4,5-5,5years (n:6) | mean (95% CI) | | | 4.56(0.33) | 18.17(13.66) | 22.33(12.8) | 4.17(6.61) |
|  | min-max | | | 4.34;4.72 | 9;28 | 15;32 | 0;8 |
| 5-6years (n:13) | mean (95% CI) | | | 5.08(0.25) | 20.69(8.92) | 24.62(8.22) | 3.92(6.76) |
|  | min-max | | | 4.84;5.25 | 12;27 | 16;32 | -2;10 |
| 5,5-6,5 years (n:12) | mean (95% CI) | | | 5.48(0.3) | 23.42(12.55) | 24.75(10.27) | 1.33(8.12) |
|  | min-max | | | 5.27;5.73 | 12;34 | 18;34 | -6;6 |
| 6-7 years (n:20) | mean (95% CI) | | | 6.02(0.33) | 23.8(9.21) | 24(12.05) | 0.2(6.72) |
|  | min-max | | | 5.76;6.24 | 15;33 | 13;33 | -7;7 |
|  | | | | | | | |
| **+Dp140** | | | **AGE** | | **NSAA T0** | **NSAA T12** | **NSAA CHANGE 12M** |
| All (n:128) | | mean (95% CI) | 5.16(1.42) | | 21.42(11.88) | 22.89(12.38) | 1.47(8.18) |
|  |  | min-max | 3.77;6.25 | | 6;33 | 5;34 | -8;15 |
| 4-5years (n:21) | | mean (95% CI) | 3.97(0.32) | | 16.76(10.16) | 19.29(10.54) | 2.52(7.16) |
|  |  | min-max | 3.77;4.22 | | 8;25 | 10;28 | -4;12 |
| 4,5-5,5years (n:18) | | mean (95% CI) | 4.56(0.32) | | 19.5(10.53) | 23.11(9.6) | 3.61(10.09) |
|  |  | min-max | 4.3;4.76 | | 8;26 | 9;31 | -2;15 |
| 5-6years (n:23) | | mean (95% CI) | 5.01(0.26) | | 20.78(7.88) | 23.09(9.51) | 2.3(7.34) |
|  |  | min-max | 4.79;5.21 | | 11;28 | 14;33 | -8;8 |
| 5,5-6,5 years (n:30) | | mean (95% CI) | 5.47(0.3) | | 22.87(11.47) | 24.17(13.19) | 1.3(8.52) |
|  |  | min-max | 5.26;5.74 | | 9;31 | 7;33 | -8;10 |
| 6-7 years (n:36) | | mean (95% CI) | 5.98(0.29) | | 24.31(12.37) | 23.69(14.44) | -0.61(6.27) |
|  |  | min-max | 5.78;6.25 | | 6;33 | 5;34 | -8;6 |
| **+Dp140 w/o steroids** | | | **AGE** | | **NSAA T0** | **NSAA T12** | **NSAA CHANGE 12M** |
| All (n:30) | | mean (95% CI) | 4.62(1.51) | | 19.8(13.73) | 20.73(11.42) | 0.93(6.37) |
|  |  | min-max | 3.77;6.12 | | 8;32 | 9;32 | -4;10 |
| 4-5years (n:13) | | mean (95% CI) | 3.97(0.33) | | 16.54(11.36) | 18.23(11.09) | 1.69(6.06) |
|  |  | min-max | 3.77;4.22 | | 8;25 | 10;26 | -4;7 |
| 4,5-5,5years (n:7) | | mean (95% CI) | 4.51(0.32) | | 19(14.27) | 20.71(12.17) | 1.71(7.9) |
|  |  | min-max | 4.33;4.74 | | 8;26 | 9;27 | -2;10 |
| 5-6years (n:3) | | mean (95% CI) | 4.94(0.09) | | 17.67(11.32) | 19.33(5.99) | 1.67(5.99) |
|  |  | min-max | 4.89;4.98 | | 11;21 | 16;22 | -1;5 |
| 5,5-6,5 years (n:2) | | mean (95% CI) | 5.37(0.22) | | 27.5(6.93) | 25.5(1.39) | -2(5.54) |
|  |  | min-max | 5.29;5.45 | | 25;30 | 25;26 | -4;0 |
| 6-7 years (n:5) | | mean (95% CI) | 6(0.24) | | 27.6(6.44) | 26.2(8.01) | -1.4(2.97) |
|  |  | min-max | 5.84;6.12 | | 24;32 | 23;32 | -3;0 |
| **+Dp140 with steroids <6m** | | | **AGE** | | **NSAA T0** | **NSAA T12** | **NSAA CHANGE 12M** |
| All (n:40) | | mean (95% CI) | 5.19(1.15) | | 20.65(11.55) | 22.77(13.11) | 2.12(8.63) |
|  |  | min-max | 4.04;6.18 | | 6;31 | 5;34 | -8;15 |
| 4-5years (n:4) | | mean (95% CI) | 4.13(0.16) | | 18(9.99) | 21.75(13.23) | 3.75(10.9) |
|  |  | min-max | 4.04;4.21 | | 13;25 | 13;28 | 0;12 |
| 4,5-5,5years (n:6) | | mean (95% CI) | 4.53(0.38) | | 19(9.44) | 23.67(5.5) | 4.67(11.27) |
|  |  | min-max | 4.3;4.75 | | 13;26 | 21;28 | -1;15 |
| 5-6years (n:8) | | mean (95% CI) | 5(0.25) | | 20.75(9.7) | 23.5(8.95) | 2.75(6.69) |
|  |  | min-max | 4.8;5.18 | | 13;26 | 17;29 | -3;8 |
| 5,5-6,5 years (n:14) | | mean (95% CI) | 5.47(0.32) | | 21.86(10.45) | 24.14(12.86) | 2.29(6.59) |
|  |  | min-max | 5.26;5.74 | | 13;28 | 14;33 | -2;8 |
| 6-7 years (n:8) | | mean (95% CI) | 5.91(0.27) | | 21(17.44) | 19.5(20.12) | -1.5(7.84) |
|  |  | min-max | 5.78;6.18 | | 6;31 | 5;34 | -8;6 |
| **+Dp140 with steroids >6m** | | | **AGE** | | **NSAA T0** | **NSAA T12** | **NSAA CHANGE 12M** |
| All (n:58) | | mean (95% CI) | 5.41(1.26) | | 22.79(10.63) | 24.09(11.93) | 1.29(8.7) |
|  |  | min-max | 3.77;6.25 | | 9;33 | 7;33 | -8;13 |
| 4-5years (n:4) | | mean (95% CI) | 3.84(0.16) | | 16.25(7.9) | 20.25(4.35) | 4(6.79) |
|  |  | min-max | 3.77;3.96 | | 12;21 | 17;22 | 1;9 |
| 4,5-5,5years (n:5) | | mean (95% CI) | 4.66(0.17) | | 20.8(6.56) | 25.8(7.51) | 5(11.84) |
|  |  | min-max | 4.53;4.76 | | 17;25 | 21;31 | -2;13 |
| 5-6years (n:12) | | mean (95% CI) | 5.02(0.29) | | 21.58(5.32) | 23.75(10.31) | 2.17(8.48) |
|  |  | min-max | 4.79;5.21 | | 17;28 | 14;33 | -8;7 |
| 5,5-6,5 years (n:14) | | mean (95% CI) | 5.49(0.3) | | 23.21(12.78) | 24(14.89) | 0.79(10.23) |
|  |  | min-max | 5.27;5.74 | | 9;31 | 7;32 | -8;10 |
| 6-7 years (n:23) | | mean (95% CI) | 6(0.3) | | 24.74(10.66) | 24.61(12.56) | -0.13(6.23) |
|  |  | min-max | 5.78;6.25 | | 11;33 | 11;32 | -8;6 |
|  | | | | | | | |
| **+Dp71** | | | **AGE** | | **NSAA T0** | **NSAA T12** | **NSAA CHANGE 12M** |
| All (n:24) | | mean (95% CI) | 5.31(1.33) | | 18.67(12.75) | 20.29(12.68) | 1.62(7.07) |
|  |  | min-max | 4;6.25 | | 8;30 | 11;33 | -3;9 |
| 4-5years (n:2) | | mean (95% CI) | 4.1(0.28) | | 8.5(1.39) | 14.5(6.93) | 6(8.32) |
|  |  | min-max | 4;4.2 | | 8;9 | 12;17 | 3;9 |
| 4,5-5,5years (n:4) | | mean (95% CI) | 4.4(0.19) | | 17.25(12.94) | 20(10.25) | 2.75(6.48) |
|  |  | min-max | 4.27;4.51 | | 10;25 | 15;25 | -1;6 |
| 5-6years (n:2) | | mean (95% CI) | 4.93(0.01) | | 21.5(18.02) | 19(16.63) | -2.5(1.39) |
|  |  | min-max | 4.93;4.94 | | 15;28 | 13;25 | -3;-2 |
| 5,5-6,5 years (n:9) | | mean (95% CI) | 5.52(0.24) | | 19.89(11.28) | 20.78(13.5) | 0.89(7.43) |
|  |  | min-max | 5.4;5.75 | | 13;30 | 11;29 | -3;6 |
| 6-7 years (n:7) | | mean (95% CI) | 6(0.26) | | 20(12.29) | 21.86(14.47) | 1.86(5.71) |
|  |  | min-max | 5.82;6.25 | | 12;27 | 12;33 | -2;6 |
| **+Dp71 w/o steroids** | | | **AGE** | | **NSAA T0** | **NSAA T12** | **NSAA CHANGE 12M** |
| All (n:5) | | mean (95% CI) | 4.94(1.76) | | 15.6(11.48) | 17.8(8.92) | 2.2(4.47) |
|  |  | min-max | 4;6.04 | | 9;25 | 12;24 | -1;5 |
| 4-5years (n:1) | | mean (95% CI) | 4(NA) | | 9(NA) | 12(NA) | 3(NA) |
|  |  | min-max | 4;4 | | 9;9 | 12;12 | 3;3 |
| 4,5-5,5years (n:2) | | mean (95% CI) | 4.46(0.12) | | 19.5(15.25) | 19.5(12.47) | 0(2.77) |
|  |  | min-max | 4.42;4.51 | | 14;25 | 15;24 | -1;1 |
| 5-6years (n:0) | | mean (95% CI) | NA(NA) | | NA(NA) | NA(NA) | NA(NA) |
|  |  | min-max | NA;NA | | NA;NA | NA;NA | NA;NA |
| 5,5-6,5 years (n:1) | | mean (95% CI) | 5.75(NA) | | 16(NA) | 19(NA) | 3(NA) |
|  |  | min-max | 5.75;5.75 | | 16;16 | 19;19 | 3;3 |
| 6-7 years (n:1) | | mean (95% CI) | 6.04(NA) | | 14(NA) | 19(NA) | 5(NA) |
|  |  | min-max | 6.04;6.04 | | 14;14 | 19;19 | 5;5 |
| **+Dp71 with steroids <6m** | | | **AGE** | | **NSAA T0** | **NSAA T12** | **NSAA CHANGE 12M** |
| All (n:7) | | mean (95% CI) | 5.55(0.88) | | 19.86(12.21) | 19.14(13.65) | -0.71(4.19) |
|  |  | min-max | 4.93;5.98 | | 13;28 | 11;28 | -3;3 |
| 4-5years (n:0) | | mean (95% CI) | NA(NA) | | NA(NA) | NA(NA) | NA(NA) |
|  |  | min-max | NA;NA | | NA;NA | NA;NA | NA;NA |
| 4,5-5,5years (n:0) | | mean (95% CI) | NA(NA) | | NA(NA) | NA(NA) | NA(NA) |
|  |  | min-max | NA;NA | | NA;NA | NA;NA | NA;NA |
| 5-6years (n:2) | | mean (95% CI) | 4.93(0.01) | | 21.5(18.02) | 19(16.63) | -2.5(1.39) |
|  |  | min-max | 4.93;4.94 | | 15;28 | 13;25 | -3;-2 |
| 5,5-6,5 years (n:2) | | mean (95% CI) | 5.62(0.19) | | 15(5.54) | 13(5.54) | -2(0) |
|  |  | min-max | 5.56;5.69 | | 13;17 | 11;15 | -2;-2 |
| 6-7 years (n:3) | | mean (95% CI) | 5.92(0.16) | | 22(11.92) | 23.33(12.6) | 1.33(2.99) |
|  |  | min-max | 5.82;5.98 | | 15;26 | 16;28 | 0;3 |
| **+Dp71 with steroids >6m** | | | **AGE** | | **NSAA T0** | **NSAA T12** | **NSAA CHANGE 12M** |
| All (n:12) | | mean (95% CI) | 5.32(1.33) | | 19.25(13.76) | 22(13.4) | 2.75(8.28) |
|  |  | min-max | 4.2;6.25 | | 8;30 | 12;33 | -3;9 |
| 4-5years (n:1) | | mean (95% CI) | 4.2(NA) | | 8(NA) | 17(NA) | 9(NA) |
|  |  | min-max | 4.2;4.2 | | 8;8 | 17;17 | 9;9 |
| 4,5-5,5years (n:2) | | mean (95% CI) | 4.34(0.18) | | 15(13.86) | 20.5(12.47) | 5.5(1.39) |
|  |  | min-max | 4.27;4.4 | | 10;20 | 16;25 | 5;6 |
| 5-6years (n:0) | | mean (95% CI) | NA(NA) | | NA(NA) | NA(NA) | NA(NA) |
|  |  | min-max | NA;NA | | NA;NA | NA;NA | NA;NA |
| 5,5-6,5 years (n:6) | | mean (95% CI) | 5.45(0.07) | | 22.17(11.19) | 23.67(12.31) | 1.5(8.38) |
|  |  | min-max | 5.4;5.48 | | 15;30 | 12;29 | -3;6 |
| 6-7 years (n:3) | | mean (95% CI) | 6.08(0.31) | | 20(14.8) | 21.33(20.96) | 1.33(8.16) |
|  |  | min-max | 5.94;6.25 | | 12;27 | 12;33 | -2;6 |

**Supplementary table 1. Age and NSAA changes for the whole population and by brain dystrophin involvement and age subgroups**
